# Effect of Substrates on the Physicochemical Properties of Li_7_La_3_Zr_2_O_12_ Films Obtained by Electrophoretic Deposition

**DOI:** 10.3390/mi14122153

**Published:** 2023-11-25

**Authors:** Efim Lyalin, Evgeniya Il’ina, Alexander Pankratov, Tamara Kuznetsova, Elena Kalinina

**Affiliations:** 1 Laboratory of Electrochemical Power Sources, Institute of High Temperature Electrochemistry, Ural Branch of the Russian Academy of Sciences, 620990 Yekaterinburg, Russia; efim.lyalin.2013@inbox.ru (E.L.); ilyina@ihte.uran.ru (E.I.); a.pankratov@ihte.uran.ru (A.P.); jess.coll@yandex.ru (T.K.); 2 Laboratory of Complex Electrophysic Investigations, Institute of Electrophysics, Ural Branch of the Russian Academy of Sciences, 620016 Yekaterinburg, Russia; 3 Department of Physical and Inorganic Chemistry, Institute of Natural Sciences and Mathematics, Ural Federal University, 620002 Yekaterinburg, Russia

**Keywords:** electrophoretic deposition, films, Li_7_La_3_Zr_2_O_12_, lithium-ion conductivity

## Abstract

Thin film technology of lithium-ion solid electrolytes should be developed for the creation of all-solid-state power sources. Solid electrolytes of the Li_7_La_3_Zr_2_O_12_ (LLZ) family are one of the promising membranes for all-solid-state batteries. LLZ films were obtained by electrophoretic deposition on Ti, Ni and steel substrates. The influence of different metal substrates on microstructure, phase composition and conductivity of the LLZ films after their heat treatment was studied. It was shown that the annealing of dried LLZ films in an Ar atmosphere leads to the transition from tetragonal modification to a low-temperature cubic structure. It was established that an impurity phase (Li_2_CO_3_) was not observed for LLZ films deposited on Ti foil after heat treatment, in contrast to films deposited on Ni and steel substrates. The highest lithium-ion conductivity values were achieved for the LLZ films annealed at 300 °C, 1.1 × 10^−8^ S cm^−1^ (at 100 °C) and 1.0 × 10^−6^ S cm^−1^ (at 200 °C).

## 1. Introduction

In the last decade, the idea of all-solid-state battery (ASSB) creation, including lithium and lithium-ion power sources, has attracted much scientific attention all over the world [1,2,3]. Advantages such as safety and the ability to operate in extreme conditions (elevated temperatures, excess pressures and aggressive environments), in contrast to traditional lithium-ion batteries, have led to increased interest in ASSBs. The use of solid electrolyte membranes in all-solid-state batteries eliminates the possibility for emergency situations when the battery’s seal is broken. Such power sources are suitable for portable equipment applications and provide the ability to power distributed devices, including distributed sensors made using micro-electromechanical systems technology. It should be noted that solid electrolytes must meet a number of requirements: high lithium-ion conductivity, low electronic conductivity, high density of ceramic membranes and compatibility with electrode materials (cathode and anode). Some inorganic solid electrolytes of the sulfide, phosphate and oxide families meet these requirements [4,5,6]; for example, lithium-ion conducting solid electrolytes of the sulfide family, such as Li_10_GeP_2_S_12_ and Li_7_P_3_S_11_. Despite the high values of lithium-ion conductivity, they have a disadvantage; they are unstable in contact with an ambient atmosphere. Solid electrolytes of the phosphate family based on Li_1+x_Al_x_Ge_2−x_(PO_4_)_3_ and Li_1+x_Al_x_Ti_2−x_(PO_4_)_3_ with NASICON-type structures possess high values of Li^+^-conductivity at room temperature (~10^−3^ S cm^−1^). However, germanium oxide is used as a precursor for Li_1+x_Al_x_Ge_2−x_(PO_4_)_3_ solid electrolyte synthesis, which has a fairly high cost and can lead to an increase in the cost of the finished device based on it. Moreover, the solid electrolytes of the sulfide and phosphate families are unstable versus an Li metal, which excludes the possibility of creating a lithium ASSB with a high energy density from them. Among oxides, the solid electrolytes of the Li_7_La_3_Zr_2_O_12_ (LLZ) family are considered one of the promising candidates for such power sources [4,5,6]. LLZ with cubic modification, stabilized by doping (for example, Ga, Al, Nb, Ta, etc.) [7,8], has high values of lithium-ion conductivity—~10^−3^ S cm^−1^ at 25 °C, as well as stability in contact with lithium metal. The undoped compound has a tetragonal structure [9] and lower values of total lithium-ion conductivity (~10^−7^ S cm^−1^). However, lithium-ion conductivity values of these two modifications become comparable at a temperature of about 300 °C [7]. According to the literature [10,11,12], LLZ also has a low-temperature cubic modification; this phase transition was observed at 250–350 °C using high-temperature X-ray diffraction. The bulk conductivity of this modification has similar values to a tetragonal LLZ (~10^−6^ S cm^−1^ at 25 °C) [11].

The transition from bulk ceramic samples of solid electrolytes to thin films (ranging from several nm to hundreds of microns) is an important task, because the thickness reduction of the solid electrolyte membrane can lead to an increase in cell electrochemical characteristics and reduction in its total resistance [13]. The development of thin-film technologies for solid-state battery creation can lead not only to an improvement of their electrical characteristics, but also to the scaling of the technology and its further commercialization. Moreover, the size of the finished device can be reduced by the use of such technology. According to the literature [7,14,15,16,17,18,19,20,21,22,23,24,25,26,27], vapor-based processes, solution and ceramic methods are the most common techniques for obtaining solid electrolytes with a garnet structure in the form of films. Among the ceramic methods, a tape casting is one of the prospective techniques of film formation because it can be easily scaled up and introduced into industrial production. Another advantage of this technique is that the formed tape, after drying, is flexible. It should be noted that a solid electrolyte powder should be mixed with organic additives (binder, plasticizer and dispersant) in a ratio whereby the film without defects is formed. These additives are necessary to form bonds, give shape and also to prevent particles from sticking together. Thus, organic components in the film give it plasticity. However, heat treatment of the solid electrolyte film can lead to an increase in its porosity due to the removal of organic components [7]. The tape casting technique can be used for the formation of solid electrolyte films of various thickness (from 10 to 100 µm). Films with thickness of less than 1 µm can be formed using solution or vapor-based processes. The choice of substrate material is one of the key factors in thin-film electrolyte formation using these techniques [7,23]. Meanwhile, flexible films can be obtained by tape casting on Mylar film, and then removed from the substrate [14,15,16,17].

MgO, Si, Al_2_O_3_, Pt, Gd_3_Ga_5_O_12_ and steel substrates are used for the formation of LLZ films using solution methods [18,19,20,21,22]. This technique is quite lengthy because it often requires the application of a large number of layers, with slow pulling of the film from the solution and careful selection of the initial components of the solution. Chen et al. [20] used a silicon substrate coated with a Pt layer for the deposition of thin films of LLZ from solution through a spin-coating process. The highest conductivity of the solid electrolytes was achieved for a film with a thickness of 0.39 μm and annealed at 600 °C (1.87 · 10^−6^ S cm^−1^). The annealing temperature increase led to a significant lithium loss due to its volatilization; however, possible interactions of LLZ with the substrate were not discussed. Tadanaga et al. [21] also used the dip-coating technique to obtain cubic LLZ films on MgO substrates. The choice of an MgO substrate was explained by the fact that annealing at 900 °C and higher leads to LLZ interaction with Al_2_O_3_, Pt and Gd_3_Ga_5_O_12_ substrates. The total conductivity of the film after its heat treatment at 900 °C was equal to 2.8 · 10^−7^ S cm^−1^ at 25 °C (Ea = 0.61 eV). Uhlenbruck et al. [22] used a steel substrate for print garnet thin films from an ink with solid electrolyte nanoparticles. It was established that the oxide layer at the steel substrate surface reacts with the sol components, which leads to unwanted reactions. Moreover, LLZ interaction with a steel substrate during annealing in the air atmosphere was observed by Bitzer et al. [19].

The formation of solid electrolyte films with a garnet structure by vapor phase deposition is also widely used [23,24,25,26,27]. Expensive equipment is one of the disadvantages of this technique. Katsui et al. [25] applied a chemical vapor deposition technique to form garnet films for the first time. The authors used Al_2_O_3_ substrates for the thin film deposition. The LLZ with tetragonal and cubic structure was obtained at 800 °C and 950 °C, respectively; the process of Al diffusion from the used substrate into the obtained thin film was detected. In work [26], a magnetron sputter process was used for Ta- and Al-doped LLZ deposition on steel foils. The deposited film of solid electrolytes reacts with the substrate at 700 °C and 800 °C. The solid electrolyte film has low conductivity values of 2 · 10^−9^ S cm^−1^. Loho et al. [27] studied the possible interaction of LLZ thin films with a Pt substrate at different temperatures and oxygen pressures. It was found that the phase formation of Li_2_PtO_3_ occurred due to LLZ interaction with the Pt substrate. Thus, the reaction of the LLZ-based compounds with the substrate material during vapor deposition processes was observed for a large number of substrates—Si, Si_3_N_4_, Al_2_O_3_, ZrO_2_, Cu, Ni, Mo, stainless steel and Pt, with the exception of MgO [23,24].

According to the literature [18,19,20,21,22,23,24,25,26,27], LLZ films can be obtained on various substrates; however, the heat treatment of the deposited film on a chosen substrate can lead to their partial interaction. In our previous work [28], the continuous ceramic coating of tetragonal LLZ on Ni and Ti substrates was successfully obtained using electrophoretic deposition (EPD). Electrophoretic deposition occurs in a liquid suspension of particles under the action of an external electric field. Particles suspended in the liquid medium acquire an excess of electrical charge as a result of solvation processes. Therefore, directed movement of particles towards the electrode with their subsequent deposition on the substrate occurs under the action of the external electric field. The mechanisms of the EPD process, the principles of choosing dispersion media and the features of regulating the suspension properties are described in multiple review works [29,30,31]. The simplicity of the technological method for carrying out the deposition process, high deposition speed and the ability to control the deposition process by changing the applied voltage and time are the main advantages of the EPD method. The EPD process is most easily carried out on conductive substrates; however, EPD can be achieved by depositing surface conductive layers to non-conductive substrates [32]. Moreover, it is possible to carry out direct EPD on porous non-conducting substrates immersed in the suspension [33,34]. Thus, the use of metal substrates for EPD significantly simplifies the deposition process, since no additional treatment of the substrates is required. We assume that the influence of substrate conductivity will be insignificant during EPD in non-aqueous media, since the bulk conductivity of the suspension will play a decisive role in the formation of films. In particular, the use of a molecular iodine additive as a charging agent influences the effective particle charge, and also leads to a significant increase in the suspension conductivity, which in many cases allows for initiation of the EPD process, even in suspensions with low zeta potential (~10 mV) [32,35,36]. The conditions of EPD were developed for obtaining LLZ films with a thickness of ~30 µm [28]. However, subsequent sintering of solid electrolyte films in the air atmosphere led to an impurity phase formation (fibers), presumably related to lithium carbonate. The thickness growth of the obtained films from 32 (initial) to 104 μm (500 °C) was observed after increases in the sample’s annealing temperature; this problem was solved by annealing films in an argon atmosphere. However, it is important to evaluate the possible interaction of the LLZ film formed by EPD with various metal substrates after its heat treatment. Commercially available metals (stainless steel, Ni and Ti) were chosen due to the possibility of subsequent commercialization of the technology, as well as their conductivity under EPD conditions. Moreover, the process of heating metals in the air atmosphere can lead to their oxidation, which can significantly affect their properties and strength.

The investigation of the microstructure, phase composition and conductivity of Li_7_La_3_Zr_2_O_12_ solid electrolyte films obtained by electrophoretic deposition on different metal substrates was the aim of the presented study.

## 2. Materials and Methods

The powder of Li_7_La_3_Zr_2_O_12_ was synthesized through a sol-gel method from La_2_O_3_ (99.9%, Vekton, St. Petersburg, Russia), Li_2_CO_3_ (99.4%, Reakhim, Moscow, Russia), and ZrO(NO_3_)_2_ · 2H_2_O (98.9%, KhimReaktivSnab, Ufa, Russia). The synthesis route was described in detail in our previous work [28]. A suspension of LLZ powder (10 g/L) in an isopropanol/acetylacetone medium with a ratio of 70/30 vol % was used for the EPD. Ultrasonic treatment (5–125 min) of the suspension was carried out in an ultrasonic bath (UZV-13/150-TH). Crystalline iodine was added to the prepared suspension of LLZ (0.4 g/L from its solution in acetylacetone) to increase the suspension conductivity and promote continuous coating formation. The properties of the suspensions and the features of their preparation are described in detail in [28].

The process of film electrophoretic deposition was carried out under constant voltage (80 V) for 4 min between the electrodes (10 mm distance). The current during EPD was measured with a UNI-TUT71E multimeter (Uni-Trend Technology, Dongguan, China), and the initial current during deposition was ~12 mA. Ni (99.9 wt%), Ti (99.6–99.9 wt %) and steel foils (10x20 mm) were used as a substrate for the LLZ deposition. Fe (66–74 wt %), Cr (17.5–20 wt %) and Ni (8–11 wt %) are the main components of the steel foil. An ST-VS-520 optical microscope (STAT, Ekaterinburg, Russia) was used for the surface study of the obtained LLZ films. Film thickness was assessed by measuring the deposited mass, taking into account the theoretical LLZ density.

Li_7_La_3_Zr_2_O_12_ coatings on different metal substrates were annealed under Ar flow (the volume fraction of carbon dioxide is no more than 0.00002%) at different temperatures (300 °C, 400 °C and 500 °C), with isothermal exposure (0.5 h) using a SUOL 2/14 V furnace (Tula-Term, Tula, Russia). The powder and annealed films were studied using X-ray diffraction (XRD). The measurements were carried out using a D-MAX-2200V diffractometer (Rigaku, Tokyo, Japan) with graphite crystal (monochromate CuKα radiation) in a 2Ө range of 15–55°. The phase composition of the annealed LLZ films on an Ni substrate was studied using Raman spectroscopy. The measurements were carried out using a Raman spectrometer U 1000 (Renishaw, England) in the range of 50−1200 cm^−1^ (spectral resolution of 1 cm^−1^).

The sample surfaces and cross-sections of the dried and annealed films were studied through scanning electron microscopy (SEM) using a MIRA3 FEG SEM (Tescan, Brno-Kohoutovice, Czech Republic). To obtain cross-sections of the LLZ films, the studied samples were placed in epoxy resin and then polished. The element distribution study of the annealed films was conducted using a JEOL JSM 5900LV SEM-Energy Dispersive Spectrometer (Oxford Instruments INCA, Richland, WA, USA).

The electrochemical impedance spectroscopy was used for the resistance determination of the obtained films using an E7-25 immittance meter (MNIPI, Minsk, Belarus) in the wide frequency and temperature ranges (0.025–1000 kHz, 25–300 °C) in air atmosphere. Pt (S = 0.095 cm^2^) was used for the electrodes [28]. The values for film thickness and electrode area were used for the total conductivity calculation. The thickness of the solid electrolytes was determined from the cross-sections of the LLZ films using an optical microscope, B100B-MS-P (AmScope, Irvine, CA, USA).

## 3. Results and Discussion

**Figure 1:**
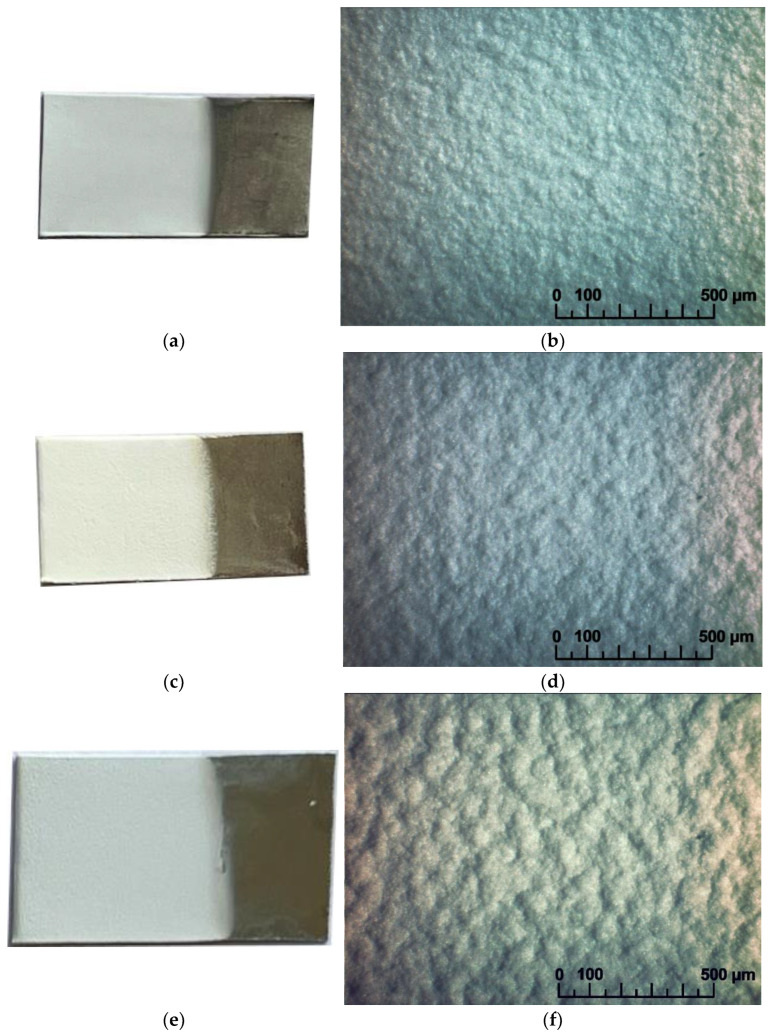
Surface of the dried LLZ films obtained by EPD on different metal substrates: (**a**,**b**)—Ti-foil; (**c**,**d**)—Ni-foil; (**e**,**f**)—steel foil.

Based on the XRD results, it can be concluded that the LLZ powder obtained by the sol-gel method was single-phase and had a tetragonal modification (I41/acd [7]). It was earlier established that the size of the ceramic grains was equal to 3–5 µm. The films of the solid electrolytes were obtained by EPD on different substrates according to the technique described in the experimental section. Continuous LLZ coatings were obtained without defects on different metal substrates under the same deposition conditions, as shown in Figure 1. It can be seen that the LLZ film obtained on steel–foil is characterized by greater roughness of the deposited layer (Figure 1e,f), which may be caused by the difference in the surface morphology of the used substrates. Despite the difference in the resistivity values of the metals (0.072, 0.068 and 0.55 µOhm · m for steel, Ni and Ti, respectively), no significant effect on the process of the film deposition was observed.

**Figure 2:**
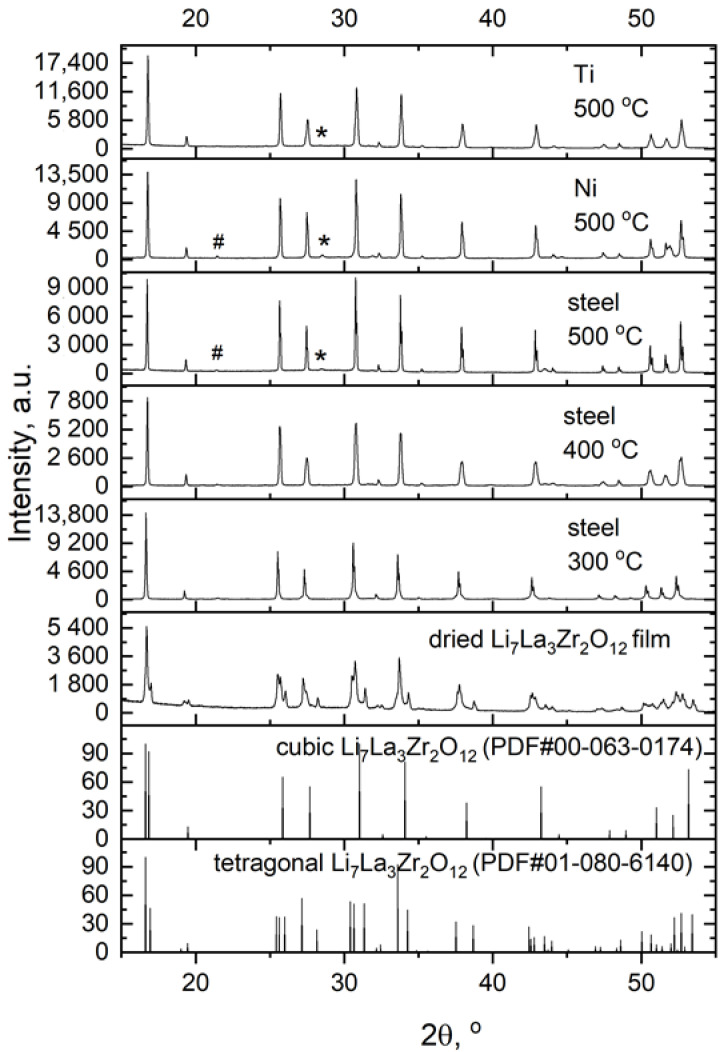
XRD patterns of the LLZ films deposited on Ni-, Ti- and steel-foils and annealed at different temperatures. *—La_2_Zr_2_O_7_ (PDF#01-071-2363). #—Li_2_CO_3_ (PDF#00-022-1141).

**Figure 3:**
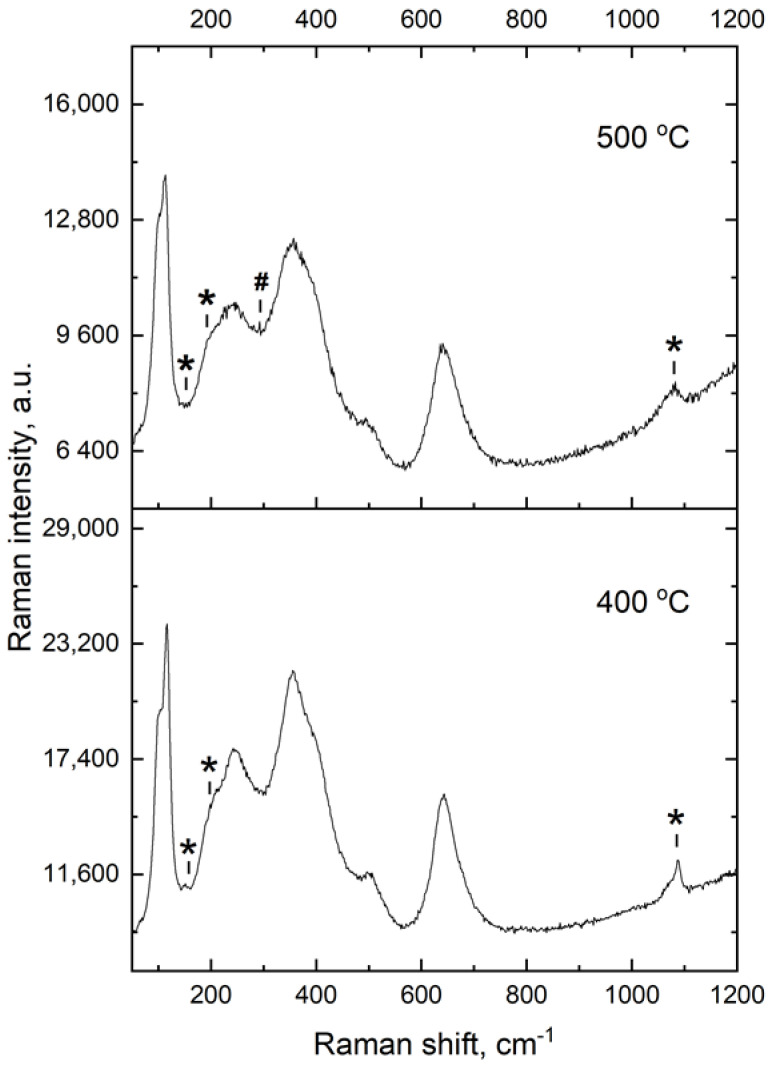
Raman spectra for the LLZ film on Ni substrate annealed at 400 and 500 °C. *—Li_2_CO_3_. #—La_2_Zr_2_O_7_.

The XRD data of the LLZ films deposited on different metal substrates after their heat treatment at different temperatures in the Ar atmosphere are presented in Figure 2. It can be seen that the annealed LLZ films have a cubic structure regardless of the substrate material. The LLZ powder was synthesized using the sol-gel method and it has a tetragonal structure; after deposition the LLZ film retains its tetragonal modification. However, heat treatment of the deposited LLZ films at 300 °C and higher leads to the transition from tetragonal modification to low-temperature cubic. This phase transition was also observed in our previous work [28], after the LLZ films annealed. It should be mentioned that according to the high-temperature XRD, phase transition from tetragonal to low-temperature cubic modification occurs from 250 to 350 °C [11,28]. Annealing of the obtained LLZ films on different metal substrates at 500 °C leads to the formation of a small amount of La_2_Zr_2_O_7_. The formation of this impurity was also observed after annealing of the LLZ film at the same temperature in the air. Moreover, small peaks related to Li_2_CO_3_ present on the XRD patterns of the LLZ films deposited on Ni and steel substrates after their annealing. However, the formation of lithium carbonate was not detected for the LLZ films deposited on a Ti substrate and annealed at different temperatures. The cubic structure of the annealed LLZ films was also confirmed by Raman spectroscopy data, as shown in Figure 3. The Raman spectra of the LLZ films deposited on Ni foil after their heat treatment at 400 and 500 °C contain the main bands at 108, 251, 360, 504 and 642 cm^−1^, which are related to the cubic modification of LLZ [37]. Moreover, there are the additional bands related to Li_2_CO_3_ (155, 205 and 1090 cm^−1^) and La_2_Zr_2_O_7_ (299 cm^−1^). It can be seen that the obtained results are in agreement with the XRD data (Figure 2).

**Figure 4:**
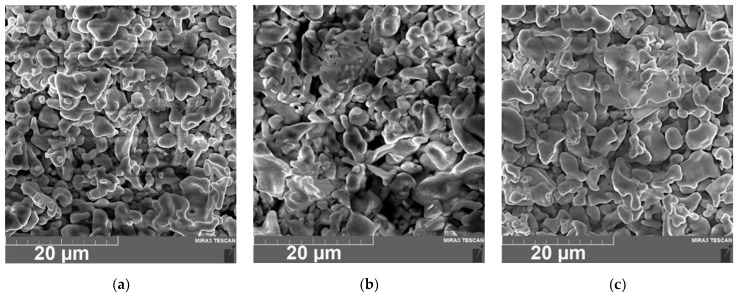
SEM micrograph of the LLZ films deposited on Ti substrate: (**a**)—dried; (**b**)—300 °C; (**c**)—500 °C.

**Figure 5:**
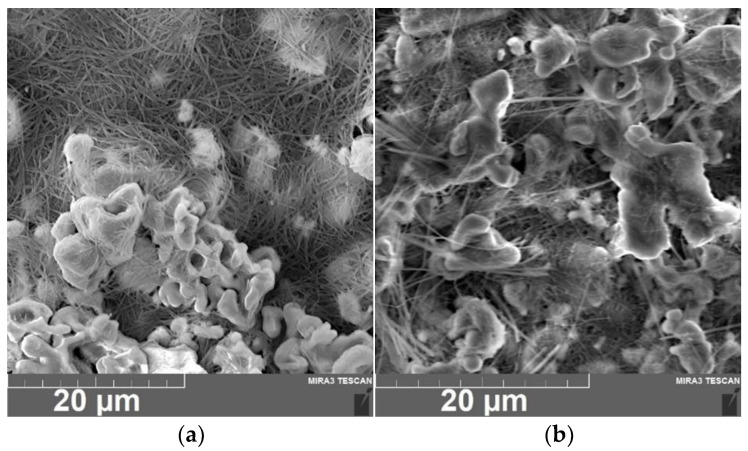
SEM micrograph of the LLZ films deposited on Ni (**a**) and steel (**b**) substrates and annealed at 300 °C.

**Figure 6:**
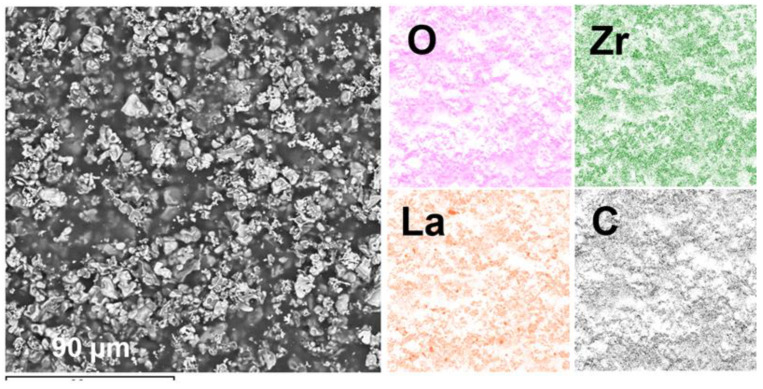
SEM micrograph of the LLZ film deposited on Ni foil and annealed at 400 °C; element distribution maps for O, Zr, La, and C.

The surfaces of the dried and annealed LLZ film deposited on a Ti substrate are presented in Figure 4. It can be seen that the dried film has a developed morphology; the LLZ grains have an irregular shape and their size varies from 1 to 5 µm (Figure 4a). The morphology does not change significantly after annealing, but the sintering of the small particles into larger ones can be observed for the film annealed at 500 °C (Figure 4c). Moreover, no lithium carbonate was observed on the LLZ films annealed in the argon atmosphere, which was detected after annealing the LLZ films in air [28]. However, on the surface of the LLZ films deposited on the Ni and steel substrates, the fibers of lithium carbonate can be observed after heat treatment (Figure 5). The presence of carbon is confirmed by an element distribution study (Figure 6). It should be noted that no interaction of LLZ with metal substrates after the samples annealing was detected. The obtained data are confirmed by the obtained XRD and Raman spectroscopy results (Figure 2 and Figure 3). It is possible that the formation of the Li_2_CO_3_ fiber phase is caused by an LLZ film interaction with trace amounts of CO_2_ in Ar, which can be observed for the LLZ deposited on the Ni and steel substrates. However, the formation of Li_2_CO_3_ in LLZ films deposited on Ti foil was not established. This may be caused by the tendency of Ti to sorb and link gases from the environment at elevated temperatures [38], which in turn leads to a CO_2_ concentration decrease near the surface of the LLZ film deposited on Ti.

**Figure 7:**
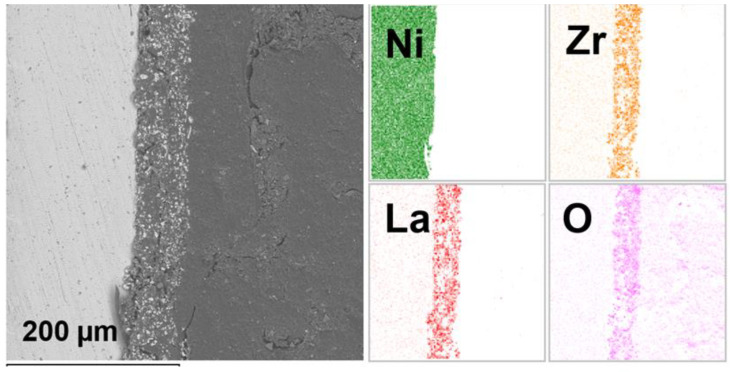
SEM micrograph of the LLZ film cross-section deposited on Ni foil and annealed at 300 °C; element distribution maps for Ni, Zr, La and O.

The cross-sections of the deposited LLZ films after their heat treatment on different metal substrates were studied using SEM with element distribution analysis. It can be seen from Figure 7 that there is clear interface between the LLZ ceramic and Ni substrate; the formation of any products from their interaction is not detected. The obtained results are similar for samples deposited on the Ti and steel substrates. The interaction of LLZ with metal substrates was not observed for all studied samples.

**Figure 8:**
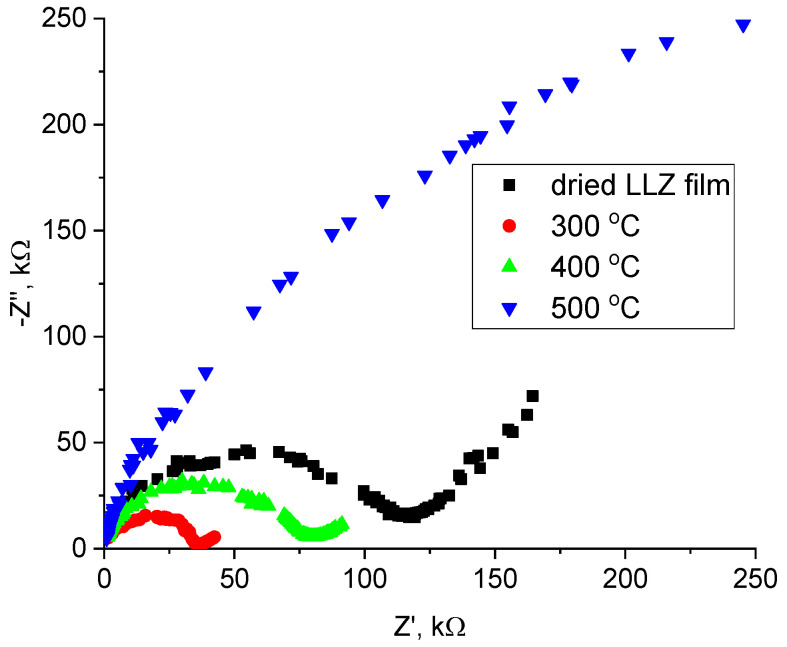
Impedance plots at 200 °C for dried and annealed LLZ films deposited on Ti substrate.

**Figure 9:**
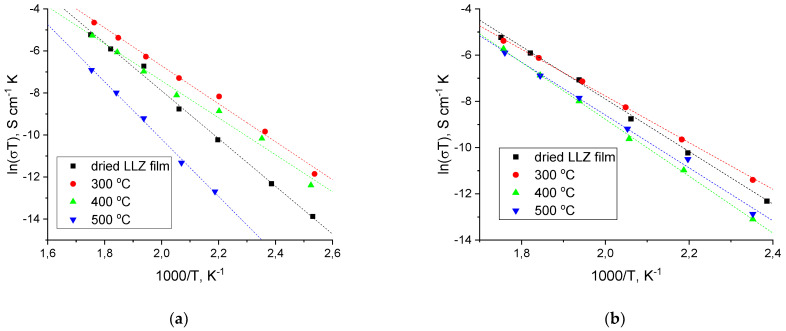
Arrhenius plots for the total conductivity of LLZ films deposited on Ti (**a**) and steel (**b**) substrates before and after their heat treatment.

The resistance of the obtained LLZ films on different metal substrates was measured using the electrochemical impedance method. The typical impedance plots at 200 °C for the LLZ films deposited on a Ti substrate are shown in Figure 8. There is only one semicircle, which starts from zero and responds to the total resistance of the LLZ membrane. The impedance plots have a similar view for all studied samples. The conductivity of the LLZ film deposited on Ti-foil increases after its annealing at 300 °C from 4.4 · 10^−10^ to 1.1 · 10^−8^ Scm^−1^ at 100 °C, while heat treatment at 500 °C leads to a significant decrease in lithium-ion conductivity (Figure 8 and Figure 9a). Such resistance growth can be caused by the impurity phase formation of La_2_Zr_2_O_7_ (Figure 2). The value of the activation energy of the deposited LLZ film is 94.4 ± 2.3 kJ mol^−1^, while the film after heat treatment at 300 °C has a lower value (74.7 ± 2.0 kJ mol^−1^). It can be assumed that sintering of the ceramic grains occurs during heat treatment of the obtained LLZ films, which leads to growth in the LLZ films conductivity and facilitates ion transport in the solid electrolyte. Therefore, the activation energy of conductivity for the annealed LLZ films decreases.

The significant conductivity growth of the solid electrolytes was not observed for LLZ films deposited on the Ni and steel substrates after their heat treatment (Figure 9b). The absence of conductivity increases after the annealing of LLZ films may be caused by the presence of impurity phases, which were detected by SEM and XRD. The thickness of the annealed films on different metal substrates was close to the dried samples and ranged from 30 to 40 µm for all studied films. The significant increase in the thickness of LLZ films from 32 µm (dried) to 104 µm (500 °C), which was detected after the sample’s heat treatment in the air atmosphere [28], was not observed. The absence of a significant increase in film thickness can be explained by the slight growth of the fiber phase on the metal foils.

The lithium-ion conductivity of the films obtained from LLZ with tetragonal structure on a Ti substrate after annealing in the Ar atmosphere possess higher values of total conductivity (1.0 · 10^−6^ S cm^−1^ at 200 °C) than after their annealing in the air atmosphere (3.6 · 10^−7^ S cm^−1^ at 200 °C [28]). However, it remains significantly lower than the lithium-ion conductivity of the LLZ bulk samples. It is possible that this problem can be solved by transition from the tetragonal modification of LLZ to doped LLZ with a cubic structure. Because some doping elements are used for the stabilization of the high-conductive structure of LLZ, in addition to structural changes, they promote ceramic sintering; this leads to density and total conductivity improvement [7,8].

## 4. Conclusions

Films of Li_7_La_3_Zr_2_O_12_ solid electrolytes with a tetragonal structure were obtained on different metal substrates using electrophoretic deposition. Continuous LLZ coatings were obtained on Ti, Ni and steel substrates under the same deposition conditions. It was established that the annealing of the obtained films in the Ar atmosphere leads to the formation of a low-temperature cubic phase. Based on the XRD, Raman spectroscopy and SEM results, it was shown that the LLZ films deposited on the Ni and steel substrates after their heat treatment contain an impurity phase—Li_2_CO_3_. Li_2_CO_3_ formation can be caused by the interaction between LLZ and trace amounts of CO_2_ in the Ar atmosphere during the film’s heat treatment. However, significant growth of film thickness during annealing in the Ar atmosphere does not occur, as was observed after the sample’s annealing in the air atmosphere. The LLZ films deposited on a Ti substrate and annealed at 300 °C are single phase and have higher conductivity values compared to the LLZ films on Ni and steel foils. The lithium-ion conductivity of the LLZ films annealed at 300 °C was equal to 1.1 · 10^−8^ S cm^−1^ at 100 °C. The obtained LLZ films can be used as lithium-ion solid electrolytes for power sources operating in medium temperatures (~300 °C). Further research will focus on the formation of solid electrolyte films by electrophoretic deposition from highly conductive, doped LLZ to create all-solid-state batteries operating at room temperature.

## Data Availability

Data are contained within the article.
